# Adult Wilms with Biphasic Pattern; A Case Report

**DOI:** 10.22088/cjim.12.0.421

**Published:** 2021

**Authors:** Mahdi Farzadnia, Mahboobe Karrabi, Hamidreza Ghorbani

**Affiliations:** 1 Department of Pathology, Mashhad University of Medical Sciences, Mashhad, Iran; 2 Kidney Transplantation Complication Research Center, Mashhad University of Medical Sciences, Mashhad, Iran

**Keywords:** Case report, Wilms tumor, Nephroblastoma, Adult

## Abstract

**Background:**

Wilms' tumor, nephroblastoma, is an extremely uncommon kidney tumor of adulthood. We reported a woman with a huge kidney mass diagnosed with nephroblastoma.

**Case presentation:**

A 39-year-old female was assessed due to right flank pain. CT scan revealed a mass measuring 128×100 mms involving the upper portion of the right kidney. The patient underwent nephrectomy, and the diagnosis of adult Wilms' tumor was confirmed based on the morphological and immunohistochemical findings.

**Conclusion:**

In adult patients with flank pain and renal mass, the diagnosis of Wilms' tumor should be pronounced in the absence of histopathologic features of renal cell carcinoma.

Wilms' tumor (nephroblastoma) is the most common pediatric renal tumor. However, it has rarely been reported during adulthood (1). Solid renal masses in adults are mostly attributed to benign cysts, renal cell carcinoma (RCC), and oncocytoma (2). Nevertheless, as stated by Kilton et al., any primary renal neoplasm of adults which represents small blue round appearance with formation of embryonal tubules in the absence of RCC histopathologic features should be evaluated for potential diagnosis of nephroblastoma and other primitive neuroectodermal tumor (3, 4). A large palpable mass in the flank without any pain is the main presenting symptom in the childhood, however, in the adults, primary symptoms include pain and hematuria (5). Lung and lever are the main sites of distant metastasis (6). We reported a woman with a huge kidney mass diagnosed with nephroblastoma.

## Case Presentation

A 39-year-old female was referred to the Department of Urology of Emam Reza Hospital, Mashhad, Iran due to persistent pain in the right flank. During the history, she had hypothyroidism which was controlled by levothyroxine. The pain had started three months ago and its intensity did not change during the day. A slight tenderness and palpable mass in the right lumbar region were detected on the physical examination without any other unremarkable findings. Routine tests were performed for the patient showing a mild microcytic hypochromic anemia, normal kidney function tests and elevated level of serum lactate dehydrogenase (753 U/L). Urine tests revealed significant red blood cell count per HPF. In an ultrasound examination, a solid mass with the size of 124×145 mm was observed in the cortex of the right upper kidney pole. 

**Figure 2:**
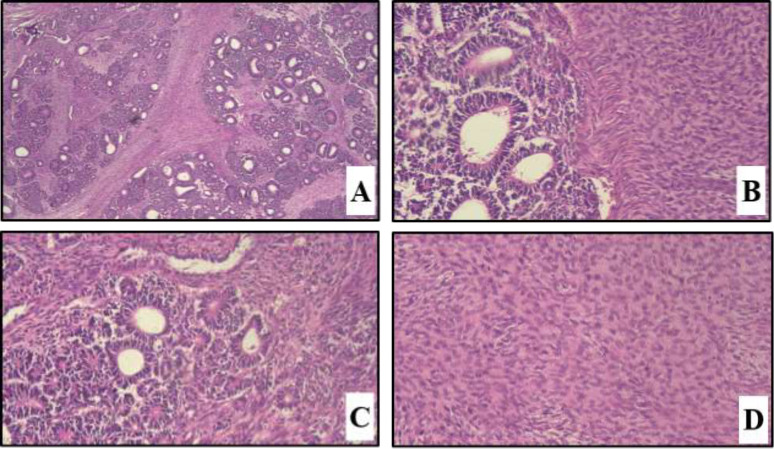
Wilms’ tumor composed of tubuloglandular and mesenchymal component. A) (40×, H&E stain). B) (200×, H&E stain).C) rosette- like structure (400×, H&E stain). D) mesenchymal stroma (400×, H&E stain)

Other abdominal and pelvic organs were reported normal. In abdominal and pelvic computed tomography (CT) scan with and without contrast, solid and hypodense mass involving the upper half right kidney was detected with approximately 128×100 mm in diameter (figure1). Also, paraaortic lymphadenopathy on the right side of the abdominal aorta was seen adjacent to the tumor, the largest was 22×11 mm in diameter. The kidney mass did not invade to surrounding structures and instead displaced liver and abdominal organs by pushing them. Other abdominal and pelvic organs were reported normal. Pulmonary and mediastinal spiral CT without contrast were performed for the patient, there was no evidence of any pulmonary or pleural metastasis. With a primary diagnosis of RCC, right radical nephrectomy was performed along with the removal of palpable pathologic lymph-nodes. Macroscopic examination shows kidney with surrounding fat weighing 1625 grams, measuring 18*13*8 cm. It has a mass with lobular outer surface and size of 16.5 * 11.5 * 7.5cm. The cut surface is creamy to white with areas of necrosis and hemorrhage surrounded by kidney with the size of 4 *3 *2 cm. The surgical margin was involved with the tumor but ureteral pelvis and sinus fat were not. In the light microscopic examination, the tumor was composed of tubular and rosette-like structures covered by simple to pseudo-stratified columnar epithelium with elongated, vesicular nuclei with acidophilic cytoplasm, occasionally with intraluminal papillary projection, enclosed in fascicular mesenchymal stroma, fibrous and hyalinized areas (figure2). Foci of necrosis was seen. Surgical margin focally involved by tumor. Blastemal element was not clearly observed in any tumor region. All resected lymph nodes were reactive without any tumoral involvement. 

**Figure 3:**
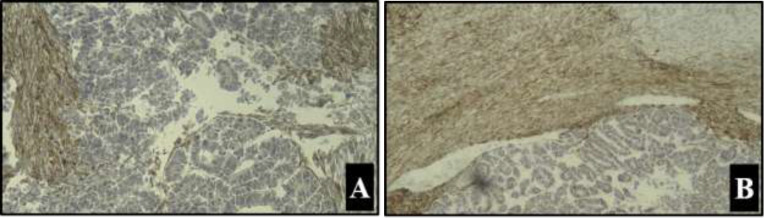
Immunohistochemical staining A) WT1 B) WT1 (repeat)

**Figure 4:**
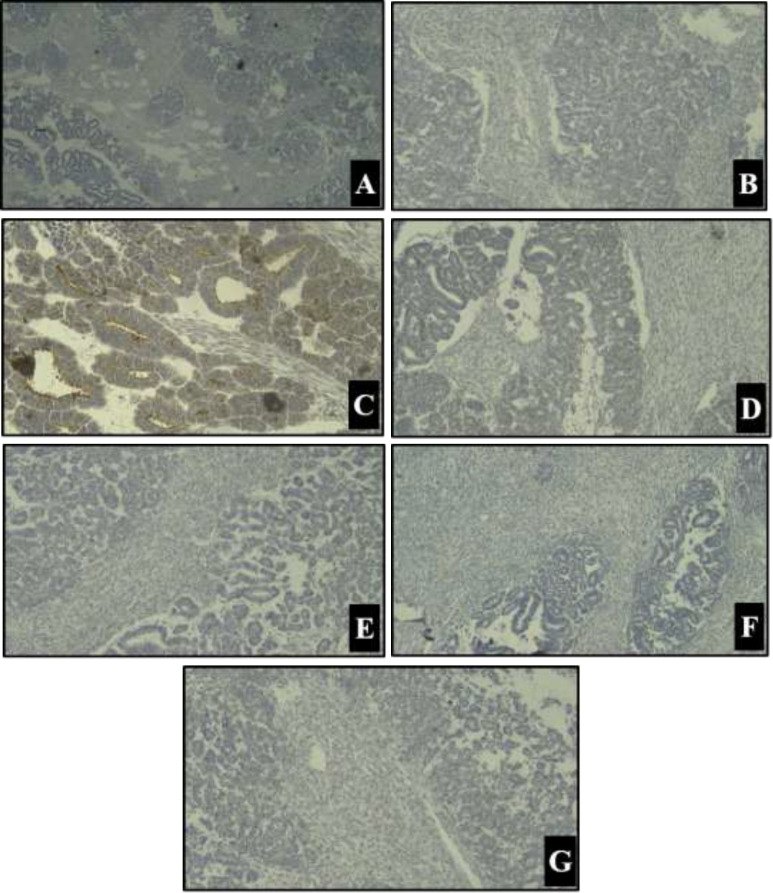
Immunohistochemical staining A) Chromogranin B) Synaptophysin C) EMA D) CK E) CD10 F) HMB45 G) CD 56

Immunohistochemistry for EMA, CK, CD56, WT1, CD10, CD99, synaptophysin, chromogranin was done. WT1 (with twice repeat) was non-specific (figure 3), EMA was positive and other markers were negative (figure 4). The final diagnosis according to histologic and immunohistochemical findings was biphasic Wilms' tumor (stage III due to surgical margin involvement).

After definitive diagnosis, patients received 6 cycles of vincristine, dactinomycin, and doxorubicin. After a two-year follow-up, the patient was disease-free. 

## Discussion

Wilms' tumor is a childhood neoplasm that most of them are under the age of 6 (7). Renal cell carcinoma is a common kidney tumor in adults, but Wilms' tumor rarely affects adults over 16 years old (8). Only less than three percent of nephroblastoma have been diagnosed during adulthood, which were difficult to diagnose and treat (8). The presence of embryonic glomerulotubular structures within the non-mature spindle stroma are essential to nephroblastoma be considered, which these are not seen usually in RCC (9). However, some of RCC subtypes have glandular and sarcomatoid or non-differentiated components, which may cause a diagnostic error (9). Wilms' diagnostic criteria based on Kilton, Matthews and Cohen criteria are:1). The tumor must be the primary neoplasm of kidney, 2) the presence of primitive blastemic spindle or round cell component, 3) formation of primary or embryonal glomerulotubular structures, 4) no region of RCC found, 5) histological confirmation, 6) age over 15 years (8)

In an Indian literature, most of the adult patients with adult Wilms' reported had a dominant blastemal component (10, 11). The differential diagnosis of adult Wilms' differs by dominant component, for example a tumor with a dominant epithelial component is indistinguishable from metanephric adenoma. Using the CD56, CD57, WT1, AMACR, CK7, CKAE1 / AE3 immunohistochemistry panel can help to differentiate these two types. In metanephric adenoma, CD56 is negative, AMACR in 10% of cases, WT1, CKAE1 / AE3 in 50% of cases are positive and CK7 is usually positive. In Wilms' tumor CD56, CD57 and WT1 in blastemal and epithelial components are positive (12). A tumor with the dominant blastemal component differential diagnosis are small blue round cell tumors such as lymphoma, rhabdomyosarcoma, or even rarely with pulmonary small cell metastasis, immature teratoma and renal cell sarcoma (8). This case due to negative CK and positive EMA staining in epithelial structure and the presence of stromal component can be distinguished from metanephric adenoma.

Most Wilms' tumor patients complain of flank pain, hematuria and many report a history of weight loss and sudden loss of consciousness. While in children, it showed more a palpable mass (8). It is a tumor in adults with large foci of necrosis and hemorrhage. About half of the patients are in stages 3 and 4 of the disease (13). In our case, necrotic areas were seen in microscopic examination, also.

There has even been a report of Wilms' tumor outside the kidney (8). This tumor can present as a non-homogeneous mass with reduced density or enhancement toward renal parenchyma, large or solid cystic mass with or without calcification on CT (9). In ultrasonography, it is a complex and large mass with large cystic components compared to renal cell carcinoma tumor, which is often a solid mass (14-16). The Wilms’ tumor angiogram shows a hypo-vascular tumor with neovascularization within the tumor, which is called “spaghetti pattern or creeping-vine”(14). In adults, WT prognosis is worse in children, and there is no explanation for it (17, 18), even on a similar stage (19). 

Adult nephroblastoma is usually diagnosed in advanced stages with large renal mass and extensive regional lymphadenopathy (20). Because of rarity of adult nephroblastoma, most treatment options are extrapolated from pediatrics with no randomized trials. In this regard, National Wilms' Tumor Study (NWTS) and other studies recommending two-three modality therapies for adult patients with nephroblastoma based on their histology and stage including surgery, chemotherapy and/or radiotherapy tumor bed boost. Less intense treatment with two drugs could be considered in patients with stage 1 and stage 2 harboring no genetic alternation (i.e. loss of heterozygosity for chromosomes 1p or 16q) (18, 21). 

In addition to histological findings, genetic and molecular studies may be necessary to gain further insight into the biology of individual tumors and may improve outcomes of aggressive treatment (20, 22-24). In conclusion in adult patients with flank pain and renal mass, Wilms' tumor should be considered. Although prognosis is worse in adults than in children, the treatment outcome is improving for adult patients with Wilms' tumor diagnosis.
